# Optimization of pneumonia CT classification model using RepVGG and spatial attention features

**DOI:** 10.3389/fmed.2023.1233724

**Published:** 2023-09-19

**Authors:** Qinyi Zhang, Jianhua Shu, Chen Chen, Zhaohang Teng, Zongyun Gu, Fangfang Li, Junling Kan

**Affiliations:** School of Medical Information Engineering, Anhui University of Chinese Medicine, Hefei, China

**Keywords:** optimization, classification model, RepVGG, attention mechanism, pneumonia

## Abstract

**Introduction:**

Pneumonia is a common and widespread infectious disease that seriously affects the life and health of patients. Especially in recent years, the outbreak of COVID-19 has caused a sharp rise in the number of confirmed cases of epidemic spread. Therefore, early detection and treatment of pneumonia are very important. However, the uneven gray distribution and structural intricacy of pneumonia images substantially impair the classification accuracy of pneumonia. In this classification task of COVID-19 and other pneumonia, because there are some commonalities between this pneumonia, even a small gap will lead to the risk of prediction deviation, it is difficult to achieve high classification accuracy by directly using the current network model to optimize the classification model.

**Methods:**

Consequently, an optimization method for the CT classification model of COVID-19 based on RepVGG was proposed. In detail, it is made up of two essential modules, feature extraction backbone and spatial attention block, which allows it to extract spatial attention features while retaining the benefits of RepVGG.

**Results:**

The model’s inference time is significantly reduced, and it shows better learning ability than RepVGG on both the training and validation sets. Compared with the existing advanced network models VGG-16, ResNet-50, GoogleNet, ViT, AlexNet, MobileViT, ConvNeXt, ShuffleNet, and RepVGG_b0, our model has demonstrated the best performance in a lot of indicators. In testing, it achieved an accuracy of 0.951, an F1 score of 0.952, and a Youden index of 0.902.

**Discussion:**

Overall, multiple experiments on the large dataset of SARS-CoV-2 CT-scan dataset reveal that this method outperforms most basic models in terms of classification and screening of COVID-19 CT, and has a significant reference value. Simultaneously, in the inspection experiment, this method outperformed other networks with residual structures.

## 1. Introduction

Pneumonia is a frequently occurring infectious disease, that can affect patients of any age. and seriously affect the life and health of patients. Currently, the primary method for assessing pulmonary conditions is by acquiring and interpreting CT lung image data, but the large number of pathological images poses a huge challenge to the work of medical personnel. The traditional classification of pneumonia mainly relies on experienced doctors, who have high requirements for the theory and experience of medical personnel, and the classification efficiency is low. At present, deep learning (1, 2) is widely used in the medical field, and artificial intelligence-assisted diagnosis is recognized by the industry.

It is important to note that the 2019 coronavirus disease (COVID-19), in addition to symptoms like fever, dry cough, and fatigue, has the potential to result in respiratory failure and life-threatening infections in extreme circumstances, making it a worldwide health concern (3). Chest CT is a non-invasive, fast, and highly sensitive method for detecting COVID-19, which can expedite the diagnosis process (4). While pneumonia caused by COVID-19 differs in some ways from other pneumonia, they share common imaging characteristics such as ground-glass opacity, patchy opacity, and consolidation. The high variability of coronaviruses, as seen in multiple outbreaks of pneumonia, has become a global medical challenge. Comparing COVID-19 CT with other pneumonia also holds research prospects for better preparedness in meeting future challenges. As a result, we compared CT scans of COVID-19 pneumonia with those of other types of pneumonia, and we created a network model that can effectively differentiate between COVID-19 pneumonia CT scans and other types of pneumonia CT scans.

Convolutional neural networks now have distinct benefits in speech recognition and image processing thanks to their special local weight sharing structure. Additionally, they are extremely important in the fields of medical picture categorization (5, 6), medical image segmentation (7, 8), medical image registration (9, 10), medical image fusion (11, 12), medical image report generation (13, 14), etc. It is common practice to classify medical images from computed tomography (CT) images using convolutional neural networks (15), X-ray films and other fields. ResNet has been cited many times in the field of medical images with good results, but its speed is limited by the depth of the network and has some limitations. This leads us to note that the RepVGG neural network, which also uses residual structures, has a faster inference time than ResNet (16).

In the work of COVID-19 and other pneumonia categorization, because there are some commonalities between viral pneumonia (1), even a small gap will lead to the risk of prediction bias, it is difficult to directly apply the current advanced network models to improve performance. Our study found that the performance of RepVGG cannot be guaranteed, despite its network structure being similar to ResNet. We observed that RepVGG downsampled the image immediately after passing through stage4, resulting in some loss of detail and found that the RepVGG network’s performance might be enhanced by superimposing a spatial attention module. Consequently, we put forth a RepVGG-based optimization strategy that includes the spatial attention block and the feature extraction backbone for the CT classification model of COVID-19. It incorporates the residual attention module (CSRA) on the classic RepVGG_b0 architecture, which allows it to extract spatial attention features while retaining the benefits of RepVGG_b0. We gave the model in our work the name RepVGG-CSRA and evaluated it using the pertinent pneumonia dataset.

The model extracts image features through the RepVGG_b0 backbone network, and then performs a convolution, and carries out two sub-sampling, respectively. The spatial information is obtained by calculation and then the score result is obtained. In addition, the introduction of a spatial attention mechanism has no significant effect on the training and inference speed of RepVGG_b0 itself. In comparison with other networks, the RepVGG-CSRA network performs better in the task of COVID-19 and other pneumonia classification.

In summary, our contributions are threefold: Firstly, we leveraged the advantages of VGG and ResNet in the classification prediction of chest CT by utilizing the properties of RepVGG. Secondly, we utilize the residual attention module utilizes the unique spatial attention of each item class in order to increase the precision of pneumonia categorization. Finally, we evaluated our model on the SARS-CoV-2 CT-scan dataset and compared its performance with the existing advanced network models VGG-16, Res-Net-50, ConvNeXt, GoogleNet, ViT, AlexNet, MobileViT, ShuffleNet, and RepVGG_b0, and achieved favorable results in the classification task.

## 2. Related works

### 2.1. CT analysis of pneumonia

**Figure 1:**
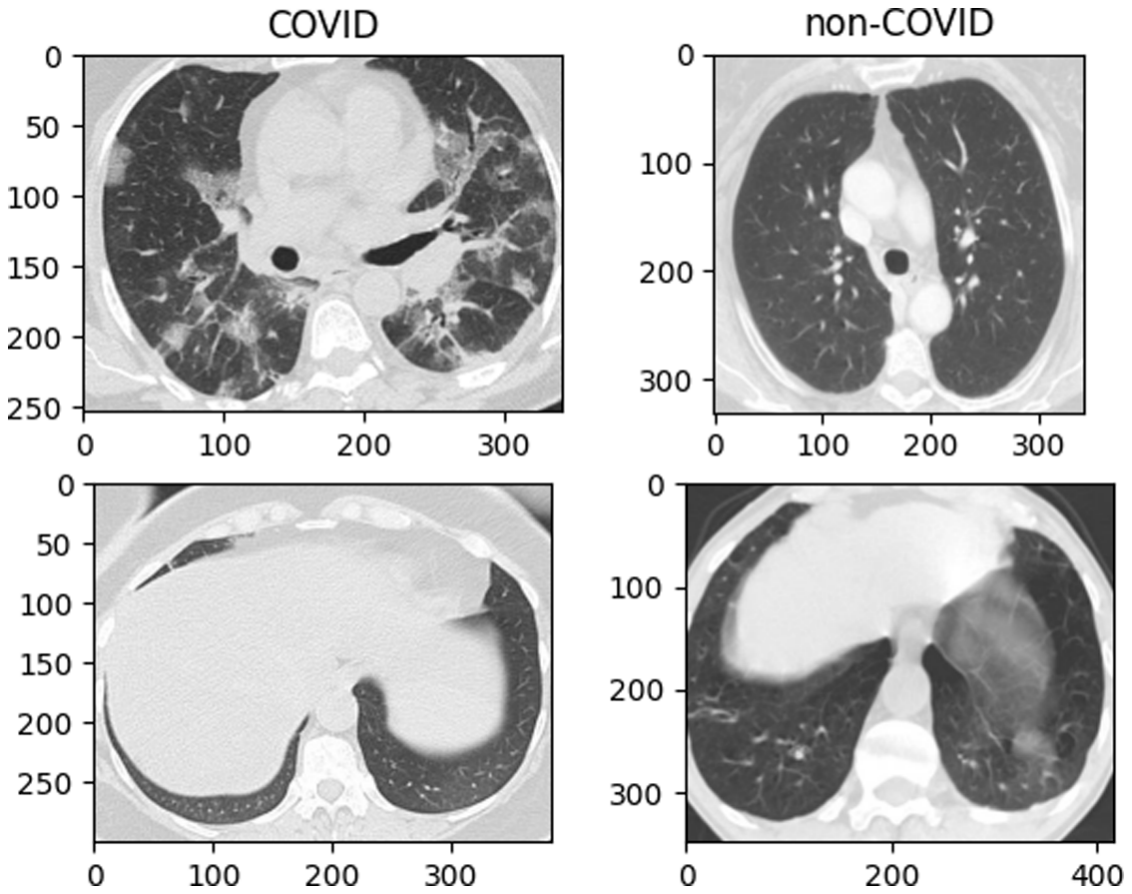
Example fundus images from the SARS-CoV-2 CT-Scan dataset.

CT images of COVID-19 show bilateral lung abnormalities, including patchy lesions and numerous scattered round lesions, whereas other pneumonia may display prominent shadows and consolidation, typically localized to specific lung regions with defined margins, are shown in Figure 1. However, the features of ground glass shadow, patch shadow and solid shadow on the image are common to most pneumonia (1, 15, 17). Because there are certain similarities between COVID-19 and other disorders in the classification work, even a slight discrepancy increases the probability of pre-diction deviation. Therefore, optimizing the network model for this CT classification task is necessary.

### 2.2. Application of deep learning in pneumonia image

Deep learning (2) is becoming increasingly significant in the area of analyzing medical images as artificial intelligence technology is developed and applied. Deep learning has showed encouraging results in the classification of medical images, as well as in the supplementary diagnosis of COVID-19. For example, Fayemiwo, MA used a deep transfer learning model to classify the COVID-19 dataset in the two-classification and three-classification tasks, trained with VGG-16, and achieved extremely high accuracy in the two-classification task (18). However, excessive stacking of convolutional blocks in the VGG network may lead to the loss of image feature information, thus reducing the accuracy. Meanwhile, problems such as gradient disappearance may occur in the training of deep neural networks. ResNet significantly reduces the issue of feature information loss by adding residual blocks to the deep convolutional neural network, thereby further improving the classification and recognition accuracy. Compared with the VGG series network, ResNet has fewer parameters and achieves better performance. Sheetal et al. proposed a new architecture using resnet50 to classify covid-19 X-ray images into three categories, with a classification accuracy of 0.979 (19, 20). Serte et al. used the ResNet-50 model to predict each CT image with greater robustness and accuracy (21). ResNet-50 used as the backbone network for the COVID-19 classification model presented by Li et al., with an AUC of 0.96 and sensitivity and specificity of 90 and 96%, respectively (22). With positive outcomes, ResNet is frequently acknowledged in the field of medical images. However, its training and inference speed is restricted by the depth of the network, and there are certain limitations. What we can know is that the convolutional neural network RepVGG, which also incorporates the residual structure, undoubtedly has a significantly faster inference time than ResNet.

### 2.3. Application of RepVGG in medical image

RepVGG proposed by Ding et al. (16), has great potential in medical image classification, which can improve accuracy while reducing computational complexity. It is an architecture for a convolutional neural network (CNN) that capitalizes on the advantages of the VGG network (23) while incorporating a ResNet-like residual structure (24). It is worth noting that CNNs have been demonstrated to outperform other image classification algorithms in terms of performance, as observed in various studies (25). RepVGG, in contrast to ResNet, maintains the straightforward VGG structure and performs better in terms of accuracy and speed. Chen et al. classified retinal OTC pictures and identified retinal disorders using the RepVGG network (26). By combining the benefits of RepVGG and Resblock, Cong et al. proposed CXR-RefineDet, which considerably increases the detection accuracy and speed (27). Kien Trang et al. used RepVGG as the backbone network and combined the VAE encoder component to developing the model. The evaluation index of the final model is greater than that of the starting model (28). This means that RepVGG could design more complex network structures with good scalability using simple stacking and connection. The method suggested in this paper, in contrast to those methods, integrates the residual attention module (CSRA) on the traditional RepVGG basic architecture to allow it to extract spatial attention features while maintaining the benefits of RepVGG, making it suitable for the classification task of a pneumonia image.

We choose the network structure of RepVGG as the backbone of the network model, which includes different sizes of residual structures and convolution kernels. There are two types of residual structures: residual structures with 1 × 1 convolution residual branches and residual structures with 1 × 1 convolution residual branches and identity residual branches.

**Figure 2:**
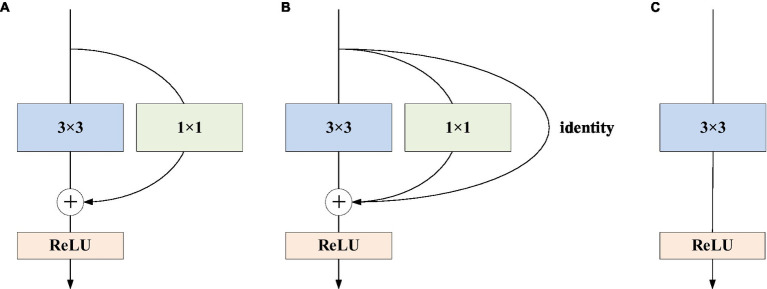
RepVGG basic structure. **(A)** The residual structures of the first layer of each stage are shown; **(B)** the residual structures of the other layers; **(C)** the inference stage’s network structure.

The training state network’s basic architecture consists of 28 layers of 3 × 3 convolution kernels divided into 5 stages with convolution numbers of 1, 4, 6, 16, and 1. Each stage’s first layer is a descending sampling of stride = (2, 2). The residual structures of the first layer of each stage are shown in Figure 2A, and the residual structures of the other layers are shown in Figure 2B, which together make up the structure of the training state network. The inference stage’s network structure is composed of 3 × 3 convolution and ReLU stacks, as illustrated in Figure 2C. To summarize, our backbone network’s structure employs repeated convolutional layers and ReLU activation functions, making the network more robust and capable of dealing with various types of medical image data changes and noise.

## 3. Our approach

**Figure 3:**
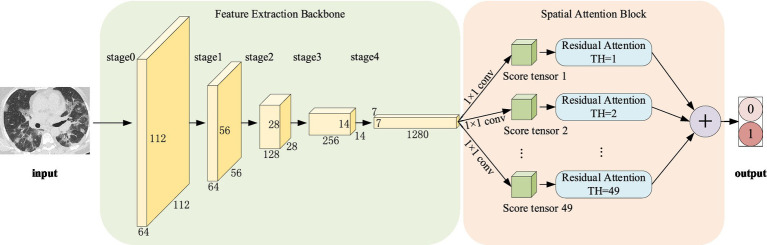
Pipeline of the proposed method.

Normally, categorization is based on contrasts or disparities between categories. It is important to note that various characteristics of pneumonia, such as numerous distributed ground glass shadows and solid shadows, were discovered to be consistent in the context of CT studies of COVID-19 and other pneumonia. However, as far as RepVGG is concerned, it is found in our study that RepVGG downsample the image immediately after passing stage 4, resulting in some details being lost. Considering this, we present a classification model that separates two classes of pneumonia using image features captured by the RepVGG backbone and the discriminative power of the different category features of CSRA. The network architecture of pneumonia CT image categorization that we created for the dataset used in this study is depicted in Figure 3. In the feature extraction backbone (FEB) mentioned above, we adopt RepVGG, and the Spatial Attention Block (SAB) is also used. FEB and SAB are the two main modules that makeup it. SAB is mostly used for classifier prediction, while FEB is primarily utilized for image feature extraction.

The algorithmic steps in the suggested approach are as follows:

**Input:** The original image of size 224 × 224 is input.

**Step 1:** Initialize the network parameters.

**Step 2:** Image uploaded in FEB, in which 0–4 stages are successively entered, and the network parameters are shown in Table 1.

**Table 1:** The network structure of RepVGG-CSRA.

Stage name	Output size	RepVGG-CSRA
Stage 0	112 × 112	Min (64, 64*1) × 1
Stage 1	56 × 56	64*1 × 4
Stage 2	28 × 28	128*1 × 6
Stage 3	14 × 14	256*1 × 16
Stage 4	7 × 7	512*2.5 × 1
Conv	7 × 7	2 × 1
Residual attention		

Step 2 involves stages 0 to 4, step 3 is convolution (conv), and steps 4 to 5 consist of residual attention.

**Step 3:** The output of stage4 is fed as input to a convolution with a 1 × 1 kernel and parameters are shown in the last penultimate row of Table 1, producing a tensor of size [1, 2, 7, 7] and divided by the norm generated by the weights of this tensor. And then we flattened them into a tensor size [1, 2, 49] as the score.

**Step 4:** Computing att_logit using spatial pooling and computing base_logit using average pooling.

**Step 5:** To obtain the network’s output, add base_logit and 0.1 times att_logit.

**Output:** Obtain the image’s score in the relevant category.

After the model has been trained on this network, the probability value of using the activation function to activate the network output to produce positive or negative results.

It is worth noting that the attention mechanism (25) is embedded in the convolutional neural network training process and is used to selectively learn and calculate the weight of input data. We incorporate a class-specific residual attention (CSRA) (29) algorithm into our network to better capture the various spatial regions occupied by items from various classes.

**Figure 4:**
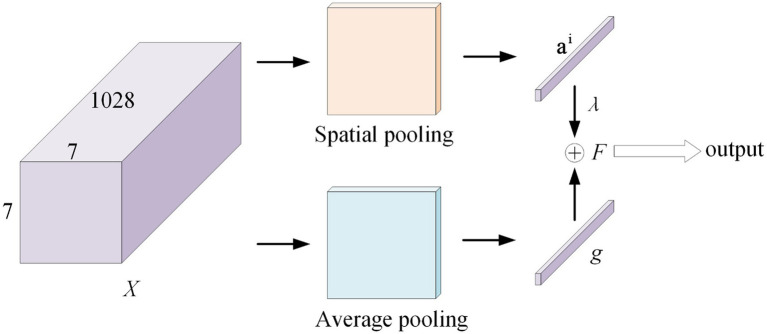
CSRA model.

As shown in Figure 4, this module first computes a spatial attention score based on features, which it then combines with its average downsampling features to generate class-specific features for each category. Figure 4 shows that *X* represents the features acquired by the backbone network, and its shape is 1,028 × 7 × 7. Placing *X* in a fully connected layer with a 1 × 1 convolution kernel. For Spatial pooling, the category-specific attention score of the *i* category and the *j*-th position is defined as Equation (1).


(1) $$s_{j}^{i}=\frac{\exp\left(TX_{j}^{T}m_{i}\right)}{\sum_{k=1}^{49}\exp\left(TX_{k}^{T}m_{i}\right)}$$

Where, the parameter of the classifier $m_{i}$ correspond to the *i*-th class is $m_{i}$∈$R^{1024}$.

Then, the specific feature vector of the *i*-th category is defined as feature tensors combined with weights, with the attention score of $S_{j}^{i}$(1 ≤ k ≤ 49) of the *i*-th category serving as the weight.


(2) $$a^{i}=\overset{49}{\underset{k=1}{\sum }}s_{k}^{i}X_{k}$$

At the same time, in the Average pooling, divide the tensor by 7 × 7 to obtain the classical average downsampling feature vector *g* of the entire image. The class-specific CSRA feature *F* of class *i* is obtained using these two vectors, as shown in Equation (3). The result is obtained by sending the feature *F* to the classifier.


(3) $$F=g+\lambda a^{i}$$

Where, λ is a hyperparameter (setting λ=0.1).

## 4. Experiments and discussion

In this section, we first described the materials we used. After that, we compare our network with RepVGG. Finally, we put the proposed model to the test, compared its performance to RepVGG’s, and evaluated its performance against other network models while screening the signs used to evaluate chest radiographs for COVID-19 pneumonia. Finally, we did a inspection experiment.

### 4.1. Datasets

To evaluate the effectiveness of our proposed model, we employ three different COVID-19 related datasets.

The experiment was primarily conducted using the SARS-CoV-2 CT-Scan dataset (30), which consists of a large collection of real patient CT scans publicly released by Eduardo Soares in 2020 from a hospital in São Paulo, Brazil. The dataset comprises 1,252 CT images of COVID-19 (infected with the virus) and 1,229 CT images of non-COVID-19 (not infected but with other lung conditions). The samples were divided into training, validation, and test sets at random in the ratios of 7:1.5:1.5.

The second dataset, which is available for download at: https://www.kaggle.com/datasets/plameneduardo/a-covid-multiclass-dataset-of-ct-scans, also originated from hospitals in São Paulo, Brazil. The dataset contains three categories: 758 images of healthy patients, 2,168 chest CT images of COVID-19 patients, and 1,247 images of other pneumonia patients (30).

The last dataset was the COVID-CT dataset (31), which comprises of 349 CT images from COVID-19 and 463 non-COVID-19 CT images. We combine the two datasets in accordance with the relevant category, exclude corrupted photos. Finally, 2,516 CT images of COVID-19 patients, 757 images of healthy patients, and 1,644 images of other pneumonia patients were obtained, which is divided into 7:1.5:1.5 for inspection experiments.

### 4.2. Evaluation metrics

We evaluated the model’s performance in COVID-19 pneumonia disease CT screening, using technical indicators such as accuracy, F1 score, Youden index, and AUC. The test set’s accuracy rate was established as the proportion of samples correctly classified by the model to all samples. The meaning of the F1 score is the harmonic average of accuracy and recall rate, calculated as shown in Equation (4).

In this study, model inference errors, such as false positives or false negatives, can be considered diagnostic errors or failure to correctly identify positive samples. Thus, the Youden index is an appropriate evaluation criterion for the model’s performance, as indicated in Equation (5). It represents the overall ability of the model to correctly identify positive and negative patients. A higher index indicates a more accurate model.


(4) $$F1\;\mathrm{score}=\frac{2*TP^{2}}{2*TP^{2}+TP*FN+TP*FP}$$

(5) $$\mathrm{Youden}\;\mathrm{index}=\frac{TN*TP-FN*FP}{\left(TP+FN\right)*\left(TN+FP\right)}$$

Positive samples predicted by the model to be in the positive class are designated as TP (true positive), negative samples predicted by the model to be in the negative class are designated as TN (true negative), negative samples predicted by the model to be in the positive class are designated as FP (false positive), and positive samples predicted by the model to be in the negative class are designated as FN (false negative).

The ROC curve’s horizontal coordinate represents the False Positive Rate, indicating the likelihood of being projected as positive when it is negative, while its vertical coordinate represents the True Positive Rate, indicating the likelihood of being expected to be positive when it is positive. The area encircled by the ROC curve and the axis is known as the AUC (Area Under Curve), as shown in Equation (6).


(6). $$AUC=\frac{\Sigma_{\mathrm{positive}}\mathrm{Ran}k_{i}-0.5*\left(M+1\right)*M}{M*N}$$

### 4.3. Experimental details

In this experiment, the train, validation and test samples of different models all adopted a unified processing scheme. The image is scaled to 256 × 256 for easy cropping to 224 × 224, and then standardized processing is carried out. The specific parameters of the training time of the network involved in the experiment are shown in Table 2. In order to meet the single variable necessary for the experiment, set the epochs of each network training process to 50 times.

**Table 2:** The parameter settings for each network model in the experiment.

Network	Optimizer	lr	Scheduler
VGG-16	Adam	1e-4	
RepVGG_b0	SGD	1e-2	
ResNet-50	Adam	1e-3	
ConvNeXt	Adam	1e-3	
GoogleNet	Adam	1e-4	
ViT	SGD	1e-3	LambdaLR
AlexNet	Adam	2e-4	
MobileViT	AdamW	2e-4	
ShuffleNet	SGD	1e-2	LambdaLR
RepVGG-CSRA	SGD	1e-2	

It is worth mentioning that the samples submitted into the model inference are guaranteed to be the same in each batch after the data is read since all models share the same inference test input order.

### 4.4. Improvements on RepVGG

**Figure 5:**
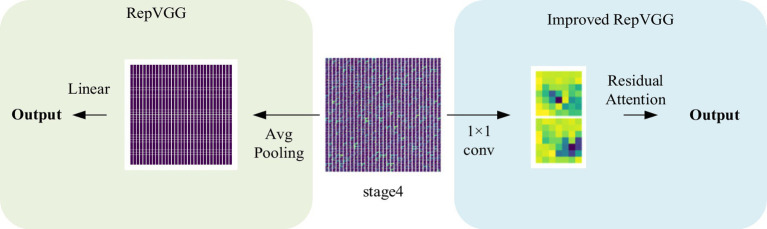
Visualization of the structure after stage4.

Although RepVGG has achieved a breakthrough for VGG, and its accuracy in pneumonia CT image classification tasks has been significantly improved compared to traditional convolutional neural networks (16), it still has some limitations. It is important to note that various characteristics of pneumonia, such as numerous distributed ground glass shadows and solid shadows, were discovered to be consistent in the context of CT study of COVID-19 and other pneu-monia. In contrast, in our study, we observed that RepVGG downsampled the image immediately after passing through stage4, resulting in some loss of detail, as shown in Figure 5. Since small changes in highly similar background tissue may indicate lesions, many fine structures cannot be ignored as they would be in natural images (1), and the RepVGG network’s approach may lack an appropriate final phase.

The experiments conducted in this study demonstrate that the integration of the CSRA spatial attention mechanism module into RepVGG results in an improved network that is capable of capturing spatial features during the last 1 × 1 convolution. By introducing the CSRA module, the network’s performance in preserving fine details that are often lost in RepVGG’s final stage is significantly enhanced. Additionally, the module allows for the establishment of correlations between each feature map.

The RepVGG-CSRA network offers a unique combination of VGG and ResNet network benefits with the added mechanism of attention feature extraction to emphasize the connections between different regions. In addition, it utilizes the heavily parameterized structure of the RepVGG network, which enhances model interpretability. Moreover, it effectively utilizes both the advantages of multi-branch and single-way models, leading to faster inference speed of the network.

### 4.5. Evaluation of our approach

**Figure 6:**
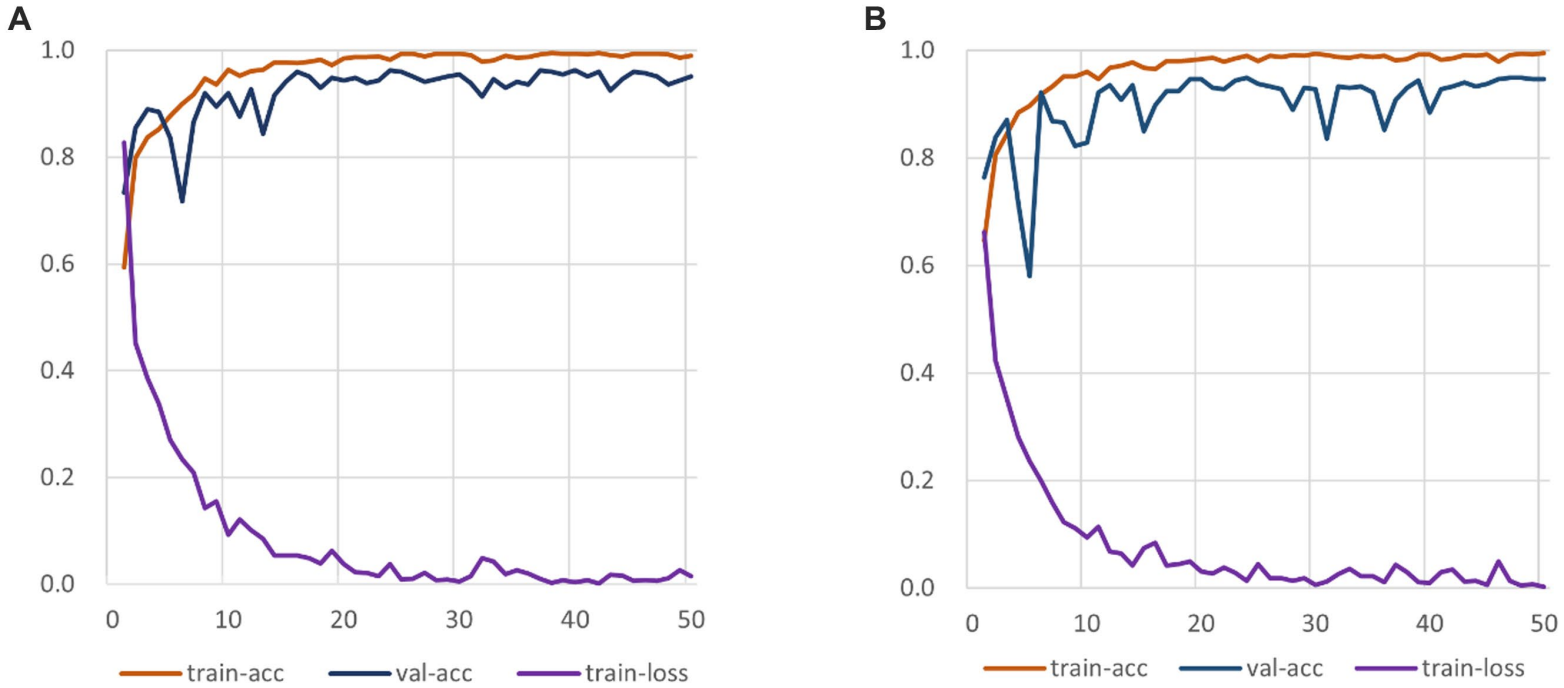
RepVGG-CSRA and RepVGG_b0 Training Process Diagram. **(A)** RepVGG-CSRA Train-ing Process Diagram; **(B)** RepVGG_b0 Training Process Diagram.

The experiment comprises three main stages: training, testing, and evaluation. The RepVGG-CSRA model is trained on both the train and validation sets during the training phase. The testing stage involves running the trained model on the independent test set to obtain the model’s prediction results. Finally, a comprehensive evaluation is conducted to assess the model’s performance on the test set. Figure 6 displays the train set accuracy, validation set accuracy, and loss value of each epoch during the RepVGG training process on the dataset used in this investigation on the SARS-CoV-2 CT-Scan dataset. The training process employs cross-entropy as a loss function to measure the discrepancy between the predicted and actual outputs. The RepVGG training process is also illustrated in Figure 6.

It is clearly observed that the RepVGG-CSRA model has a higher accuracy on the validation set than the RepVGG_b0 model over 50 epochs. At the same time, it can be observed that there is not much difference between the train-acc and val-acc curves, indicating that the fitting of our model is relatively good, even better than that of RepVGG_b0 model. When the epoch is smaller than 10, the loss values of both decline quickly, and as the number of iterations increases, they finally converge. We continue to observe the changes of the loss curve and find that both converges when epoch is about 25. To our satisfaction, the new model outperforms the RepVGG model in terms of convergence speed and train-loss smoothness.

**Figure 7:**
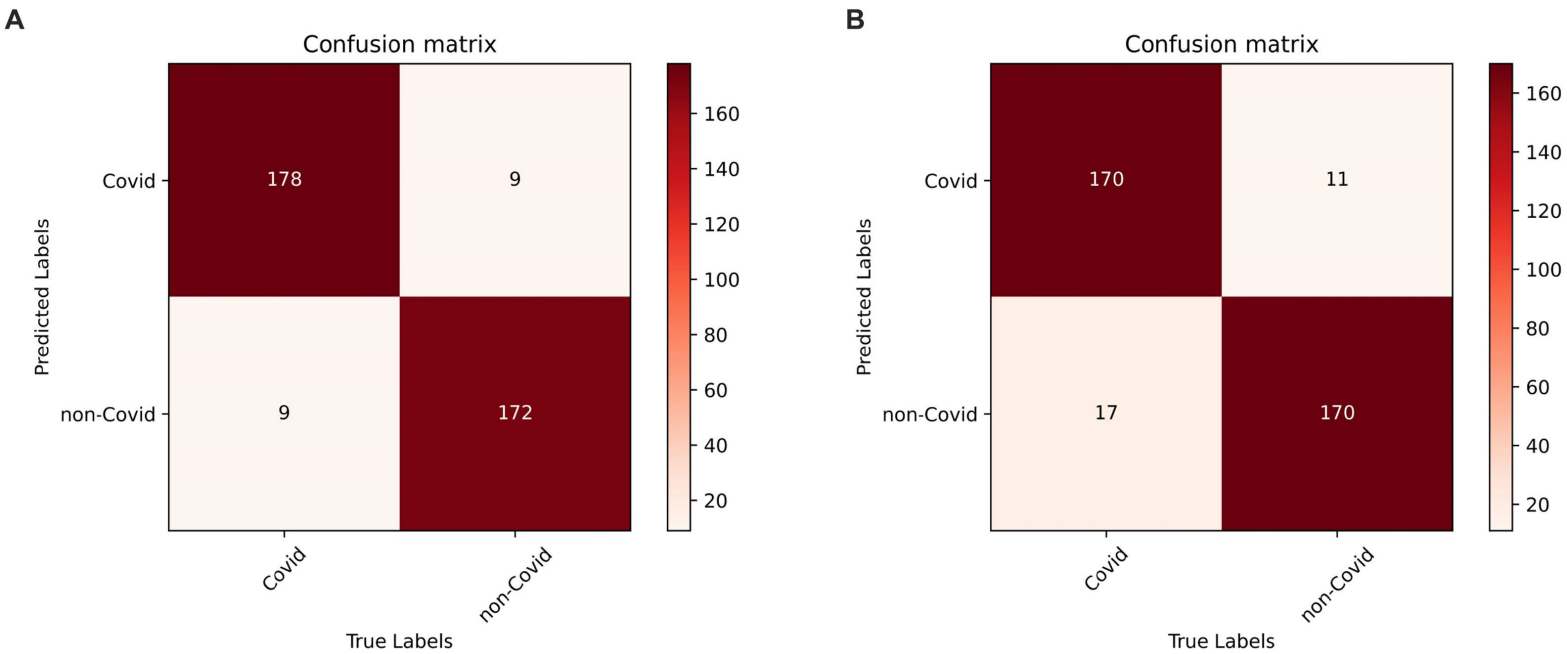
RepVGG-CSRA and RepVGG_b0 confusion matrix diagram. **(A)** RepVGG-CSRA confu-sion matrix diagram; **(B)** RepVGG_b0 confusion matrix diagram.

In the prediction classification model, higher prediction accuracy is desirable. To assess the effectiveness of the RepVGG-CSRA and RepVGG_b0 models, confusion matrices were generated based on the results obtained on the test set, as shown in Figure 7. In this confusion matrix, higher values of TP and TN represent better results, while corresponding FP and FN values should be lower. This figure shows that although there were some errors in the inference results, most of the data is concentrated on the diagonal seats, indicating that RepVGG_b0 and its improved model are suitable for this dataset. The two models’ relative accuracy on the test set is 0.951 and 0.924. Notably, the accuracy of the RepVGG-CSRA model is 2.7 percentage points higher than the RepVGG_b0 model. To comprehensively assess the performance enhancement of the RepVGG-CSRA model, we considered five indicators: precision, recall, specificity, F1-score, and Youden index.

Tables 3, 4 present the results of our evaluation on the RepVGG-CSRA and RepVGG_b0 models for the classification task. Notably, these models show consistent patterns in terms of their Specificity, Precision, and Recall values. The calculated Youden index values also reveal a well-balanced dataset, with both classes having similar performance. Overall, Tables 3, 4 demonstrate that the RepVGG-CSRA model outperforms the RepVGG_b0 model by more than 1 percentage point in all categories. Particularly, the Youden index of the RepVGG-CSRA model is significantly greater than that of the RepVGG_b0 model, indicating that our approach is more effective and authentic for screening COVID-19 pneumonia from CT images.

**Table 3:** Indicator calculation results of RepVGG-CSRA model.

Class	Precision	Recall	Specificity	F1 score	Youden index
COVID	0.952	0.952	0.950	0.952	0.902
Non-COVID	0.950	0.950	0.952	0.950	0.902

**Table 4:** Indicator calculation results of RepVGG_b0 model.

Class	Precision	Recall	Specificity	F1 score	Youden index
COVID	0.939	0.909	0.939	0.924	0.848
Non-COVID	0.909	0.939	0.909	0.924	0.848

### 4.6. Compared to other approaches

Using the evaluation criteria and experimental setup previously described, we compared the network model proposed in this paper to existing convolutional neural network models during the inference and evaluation stages. We performed unified prediction on the test set and plotted the confusion matrix to calculate the accuracy, precision, recall, specificity, F1 score, and Youden index results for predicting COVID-19 positive categories on the SARS-CoV-2 CT-Scan dataset. The outcomes are presented in Table 5.

**Table 5:** Evaluation results for each model.

Model	Precision	Recall	Specificity	Accuracy	F1 score	Youden index
VGG-16	0.908	0.947	0.901	0.924	0.927	0.848
RepVGG_b0	0.939	0.909	0.939	0.924	0.924	0.848
ResNet-50	0.935	0.925	0.934	0.929	0.930	0.859
ConvNeXt	0.840	0.840	0.834	0.837	0.840	0.674
GoogleNet	0.929	**0.973**	0.923	0.948	0.950	0.896
ViT	0.955	0.791	**0.961**	0.875	0.865	0.752
AlexNet	0.927	0.947	0.923	0.935	0.937	0.87
MobileViT	0.888	0.845	0.890	0.867	0.866	0.735
ShuffleNet	**0.961**	0.920	**0.961**	0.940	0.940	0.881
**RepVGG-CSRA**	0.952	0.952	0.950	**0.951**	**0.952**	**0.902**

Bold Values indicates the best one of the models on each metric.

Table 5 provides evidence for this that RepVGG-CSRA has the highest accuracy compared with other experimental models. The precision is not obvious advantage, but the F1 score calculated using the recall rate also achieved the highest value, which is 11.2% higher than the ConvNeXt model, indicating that the model had a relatively good performance in the detection of COVID-19. In addition, the Youden index calculated for each model found that the value of the RepVGG-CSRA network model jumped from 80 to 90%, further indicating that our model is better in screening COVID-19 and has a certain degree of authenticity.

**Figure 8:**
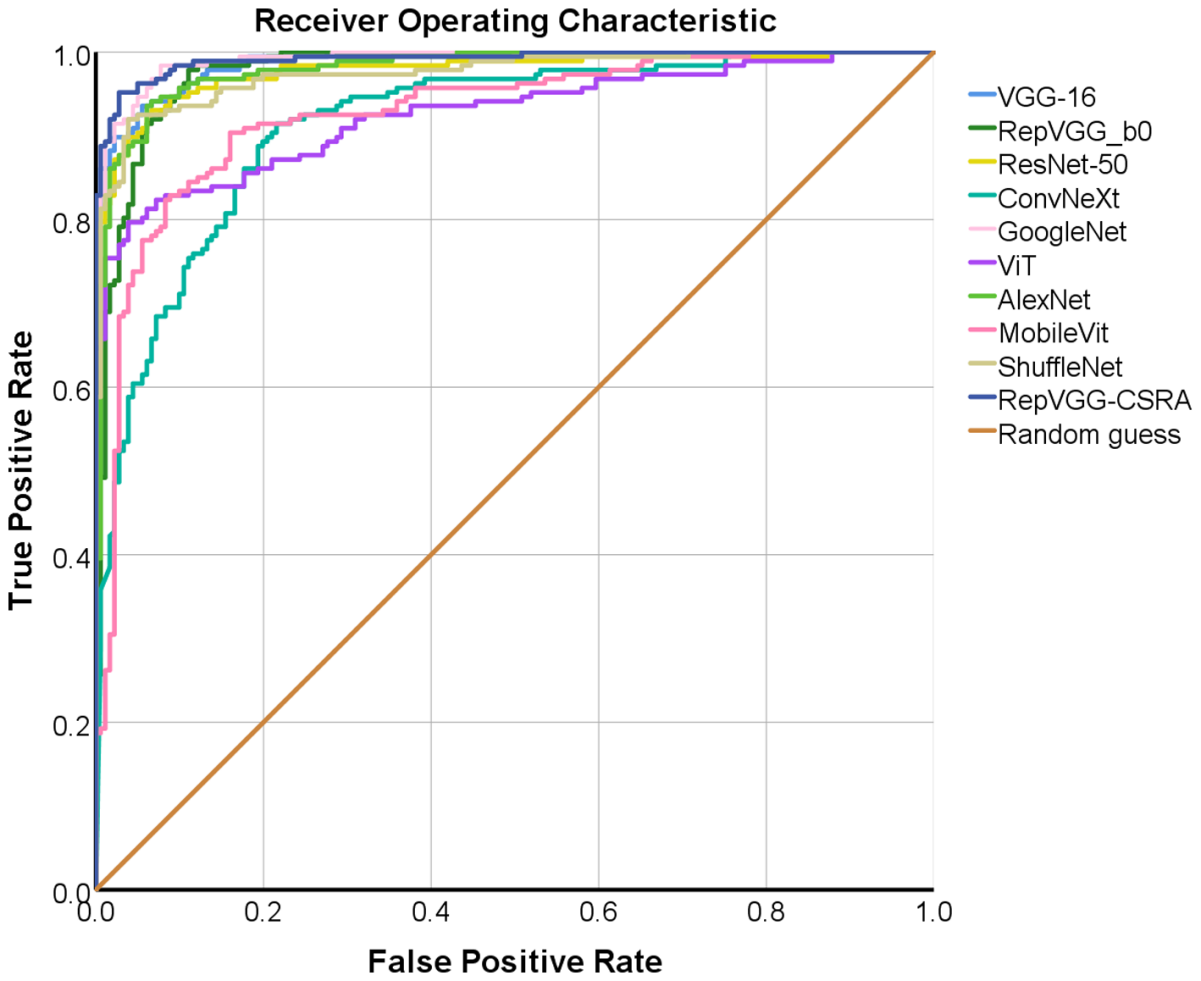
The ROC per category for classification on the pneumonia dataset.

As described above, ROC curve is a curve reflecting the relationship between sensitivity and specificity. Figure 8 displays the test set with the ROC curves for each model. The precision increases as the abscissa gets nearer to zero. The larger the ordinate, the better the accuracy. By observing the graph, it can be found that there are two curves closest to the coordinate (0, 1.0), which are GoogleNet model and RepVGG-CSRA model. Table 6 shows the calculated curve area results. It can be seen from the numerical value that half of the experimental models, including RepVGG-CSRA model, have AUC value>0.95, which is close to perfect classifier. In addition, compared with the GoogleNet model on this metric, our model exceeds the result of most models by 1 percentage point, and the error is relatively low, indicating that it has certain advantages in the classification of SARS-CoV-2 CT scan dataset. Based on the above evaluation, our model has performed the best according to a lot of indicators.

**Table 6:** AUC calculation results.

Variable	Area	Standard error	Asymptotic 95% confidence interval	
			Lower limit	Superior limit
VGG-16	**0.986**	0.005	0.977	0.996
RepVGG_b0	0.978	0.007	0.965	0.991
ResNet-50	0.978	0.007	0.964	0.992
ConvNeXt	0.915	0.014	0.886	0.943
GoogleNet	**0.991**	0.003	0.984	0.997
ViT	0.926	0.014	0.899	0.953
AlexNet	**0.980**	0.006	0.969	0.992
MobileViT	0.927	0.014	0.900	0.955
ShuffleNet	0.975	0.007	0.961	0.989
**RepVGG-CSRA**	**0.991**	0.004	0.984	0.998

The top three values in the row in each index are in bold.

Based on the comprehensive evaluation metrics, The RepVGG-CSRA model, among the experimental models, was found to have the best performance for detecting COVID-19 using CT scan datasets. The RepVGG-CSRA model has advantages in the SARS-CoV-2 CT-Scan dataset screening due to its high accuracy, F1 score, AUC value and Youden index.

### 4.7. Inspection experiment

In this paper, we conduct tests on the second and third datasets, in order to test the generality of our method. It should be noted that the dataset’s class imbalance may cause the model to overemphasize the larger number of categories and undervalue the smaller number of categories, which could have an impact on the model’s performance.

We perform adjustment augmentation on the training set. The steps of the specific enhancement operations are as follows:

- In the Covid category, 30 percent of the data were randomly selected for brightness enhancement, resulting in a total of 2,288 images.
- In the Healthy category, all the data underwent brightness enhancement, contrast enhancement, and hue adjustment, resulting in a total of 2,116 images.
- In the Others category, all the data underwent brightness enhancement, resulting in a total of 2,300 images.

**Figure 9:**
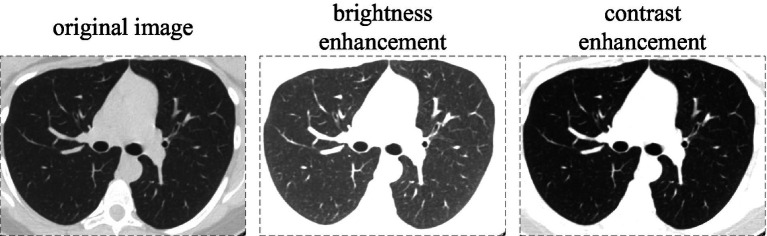
Brightness and contrast enhancements are applied to the images in the dataset.

The aforementioned parameters for brightness enhancement, saturation enhancement, and color adjustment are 1.5, 1.5, and 0.5, respectively, with part of this shown in Figure 9.

We retained the details of the above experiments, which involved training on the training and validation sets of the new dataset and testing on the test set, with the obtained test results shown in Table 7. We specifically focus on comparing models with residual network structures. The accuracy of RepVGG-CSRA improves, demonstrating relatively superior performance compared to other models with residual structures.

**Table 7:** The evaluation results are tested for each model.

Model	Class	Precision	Recall	Specificity	F1 score	Youden index	Accuracy
RepVGG_b0	0	0.771	0.832	0.742	0.800	0.574	0.7595
	1	0.778	0.67	**0.965**	0.720	0.635	
	2	0.732	0.69	0.874	0.710	0.564	
ResNet-50	0	0.825	0.827	0.817	0.826	0.644	0.7649
	1	0.721	0.652	0.953	0.685	0.605	
	2	0.694	0.722	0.841	0.708	0.563	
ShuffleNet	0	0.786	**0.88**	0.75	**0.830**	0.63	0.6426
	1	0.745	0.661	0.958	0.700	0.619	
	2	0.775	0.673	0.902	0.720	0.575	
**RepVGG-CSRA**	0	**0.836**	0.814	0.833	0.825	0.647	**0.7663**
	1	0.669	0.809	0.926	0.732	**0.735**	
	2	0.714	0.673	0.866	0.693	0.539	

Class 0 represents Covid; Class 1 represents Healthy; Class 2 represents Others. Bold Values indicates the best one of the models on each metric.

## 5. Conclusion

Patients’ lives and health are significantly impacted by the broad and prevalent infectious disease known as pneumonia. Particularly in recent years, the COVID-19 outbreak has caused a sharp rise in the number of confirmed cases of epidemic transmission. Artificial intelligence technology is commonly employed to better serve the patient and doctor populations because it successfully decreases the influence of human error.

The learning ability of the improved model, RepVGG-CSRA, has been enhanced to some extent. On the training set and verification set, compared with RepVGG, our fitting speed is faster and the results are better. This model incorporates the residual attention module into the classic RepVGG basic architecture, which allows it to extract spatial attention features while retaining the benefits of RepVGG.

Our experiments demonstrate that a network model improved upon RepVGG offers superior screening on the SARS-CoV-2 CT-scan dataset compared to traditional models. RepVGG-CSRA can detect the majority of COVID-19 lung images on the test set, with calculated recall and specificity above 95%. In addition, the calculated values of Accuracy, F1 score and Youden index reached the optimum in the experiment. The most noteworthy aspect of this work is that our improved model outperformed many network models in evaluating various indicators on the test set. In addition, when the other two datasets are fused, RepVGG-CSRA achieves the best performance among the currently popular residual network structures, which is 1 percentage point higher than the pre-improvement RepVGG. However, its performance is not as good on the other two datasets as it is on the SARS-CoV-2 dataset.

Computer-aided diagnosis requires accurate classification screening, for which traditional network models have yielded excellent results. However, further research is needed to determine whether this model can perform well on other image datasets and whether there are certain usage restrictions. This also reflects the unpredictability of artificial intelligence in medical diagnosis, therefore intelligent diagnostic tools are only useful as tools to aid medical professionals and cannot be utilized for patient self-examination.

## Data availability statement

The original contributions presented in the study are included in the article/supplementary material, further inquiries can be directed to the corresponding authors.

## Author contributions

QZ, ZG, JK, and JS designed the study. QZ and ZG performed the design and implementation of the work. QZ, JS, and CC wrote the first draft of the article, edited, and revised the article. ZT helped to review and improve the manuscript. FL and CC scrutinized the data and experiments. All authors contributed to and have approved the final version of the article.

## Funding

This work was supported by the funding for the Provincial First-Class Professional Construction Site Project of the Information Management and Information System Specialty of Anhui University of Traditional Chinese Medicine, the Key Project of Natural Science Foundation of Anhui Province (Nos. KJ2020A0394 and 2022AH050475), and the Key Project of Anhui University of Traditional Chinese Medicine’s Natural Science Foundation (No. 2021zrzd12).

## Conflict of interest

The authors declare that the research was conducted in the absence of any commercial or financial relationships that could be construed as a potential conflict of interest.

## Publisher’s note

All claims expressed in this article are solely those of the authors and do not necessarily represent those of their affiliated organizations, or those of the publisher, the editors and the reviewers. Any product that may be evaluated in this article, or claim that may be made by its manufacturer, is not guaranteed or endorsed by the publisher.

## References

[1] Wang S Kang B Ma J Zeng X Xiao M Guo J. A deep learning algorithm using CT images to screen for Corona virus disease (COVID-19). Eur Radiol. (2021) 31:6096–04. doi: 10.1007/s00330-021-07715-1, PMID: 33629156 PMC7904034

[2] LeCun Y Bengio Y Hinton G. Deep learning. Nature. (2015) 521:436–4. doi: 10.1038/nature14539 26017442

[3] Huang C Wang Y Li X Ren L Zhao J Hu Y. Clinical features of patients infected with 2019 novel coronavirus in Wuhan China. The Lancet. (2020) 395:497–6. doi: 10.1016/S0140-6736(20)30183-5 PMC7159299 31986264

[4] Ai T Yang Z Hou H Zhan C Chen C Lv W. Correlation of chest CT and RT-PCR testing for coronavirus disease 2019 (COVID-19) in China: a report of 1014 cases. Radiology. (2020) 296:E32–40. doi: 10.1148/radiol.2020200642, PMID: 32101510 PMC7233399

[5] Lakhani P Sundaram B. Deep learning at chest radiography: automated classification of pulmonary tuberculosis by using convolutional neural networks. Radiology. (2017) 284:574–2. doi: 10.1148/radiol.2017162326, PMID: 28436741

[6] Masquelin AH Cheney N Kinsey CM Bates JHT. Wavelet decomposition facilitates training on small datasets for medical image classification by deep learning. Histochem Cell Biol. (2021) 155:309–7. doi: 10.1007/s00418-020-01961-y, PMID: 33502624 PMC7957953

[7] Müller D Kramer F. MIScnn: a framework for medical image segmentation with convolutional neural networks and deep learning. BMC Med Imaging. (2021) 21:12–1. doi: 10.1186/s12880-020-00543-7, PMID: 33461500 PMC7814713

[8] You H Yu L Tian S Cai W. DR-net: dual-rotation network with feature map enhancement for medical image segmentation. Comp Intell Syst. (2021) 8:611–3. doi: 10.1007/s40747-021-00525-4

[9] Song L Liu G Ma M. TD-net: unsupervised medical image registration network based on transformer and CNN. Appl Intell. (2022) 52:18201–9. doi: 10.1007/s10489-022-03472-w

[10] Haskins G Kruger U Yan P. Deep learning in medical image registration: a survey. Mach Vis Appl. (2020) 31:1–18. doi: 10.1007/s00138-020-01060-x

[11] Kaur M Singh D. Multi-modality medical image fusion technique using multi-objective differential evolution based deep neural networks, journal of ambient intelligence and humanized. Computing. (2021) 12:2483–93. doi: 10.1007/s12652-020-02386-0, PMID: 32837596 PMC7414903

[12] Li Y Zhao J Lv Z Li J. Medical image fusion method by deep learning. Int J Cogn Comput Eng. (2021) 2:21–9. doi: 10.1016/j.ijcce.2020.12.004

[13] Zhang Y Wang X Xu Z Yu Q Yuille A Xu D. When radiology report generation meets knowledge graph. Proc AAAI Conf Artif Intell. (2020) 34:12910–7. doi: 10.1609/aaai.v34i07.6989

[14] Liu F Wu X Ge S Fan W Zou Y, Exploring and distilling posterior and prior knowledge for radiology report generation. Proceedings of the IEEE/CVF Conference on Computer Vision and Pattern Recognition (2021).

[15] Lei K Mardani M Pauly JM Vasanawala SS. Wasserstein GANs for MR imaging: from paired to unpaired training. IEEE Trans Med Imaging. (2020) 40:105–5. doi: 10.1109/TMI.2020.3022968, PMID: 32915728 PMC7797774

[16] Ding X Zhang X Ma N, et al Making vgg-style convnets great again. (2021). Proceedings of the IEEE/CVF Conference on Computer Vision and Pattern Recognition Repvgg Available at: https://openaccess.thecvf.com/content/CVPR2021/html/Ding_RepVGG_Making_VGG-Style_ConvNets_Great_Again_CVPR_2021_paper.html

[17] Wong HYF Lam HYS Fong AHT Leung ST Chin TWY Lo CSY. Frequency and distribution of chest radiographic findings in patients positive for COVID-19. Radiology. (2020) 296:E72–8. doi: 10.1148/radiol.2020201160, PMID: 32216717 PMC7233401

[18] Fayemiwo MA Olowookere TA Arekete SA Ogunde AO Odim MO Oguntunde BO. Modeling a deep transfer learning framework for the classification of COVID-19 radiology dataset. PeerJ Comp Sci. (2021) 7:e614. doi: 10.7717/PEERJ-CS.614, PMID: 34435093 PMC8356654

[19] Zhang X Zhou X Lin M Sun J Shufflenet An extremely efficient convolutional neural network for mobile devices. Proceedings of the IEEE conference on computer vision and pattern recognition. (2018). Available at: https://openaccess.thecvf.com/content_cvpr_2018/html/Zhang_ShuffleNet_An_Extremely_CVPR_2018_paper.html

[20] Pourdarbani R Sabzi S Dehghankar M Rohban MH Arribas JI. Examination of lemon bruising using different CNN-based classifiers and local spectral-spatial hyperspectral imaging. Algorithms. (2023) 16:113. doi: 10.3390/a16020113

[21] Serte S Demirel H. Deep learning for diagnosis of COVID-19 using 3D CT scans. Comput Biol Med. (2021) 132:104306. doi: 10.1016/j.compbiomed.2021.104306, PMID: 33780867 PMC7943389

[22] Li L Qin L Xu Z Yin Y Wang X Kong B. Using artificial intelligence to detect COVID-19 and community-acquired pneumonia based on pulmonary CT: evaluation of the diagnostic accuracy. Radiology. (2020) 296:E65–71. doi: 10.1148/radiol.2020200905, PMID: 32191588 PMC7233473

[23] Simonyan K Zisserman A. Very deep convolutional networks for large-scale image recognition. arXiv:1409.1556 (2014). doi: 10.48550/arXiv.1409.1556,

[24] He K Zhang X Ren S Sun J. (2016). Deep residual learning for image recognition. Proceedings of the IEEE Conference on Computer Vision and Pattern Recognition Available at: https://openaccess.thecvf.com/content_cvpr_2016/html/He_Deep_Residual_Learning_CVPR_2016_paper.html

[25] Gu J Wang Z Kuen J Ma L Shahroudy A Shuai B. Recent advances in convolutional neural networks. Pattern Recogn. (2018) 77:354–7. doi: 10.1016/j.patcog.2017.10.013

[26] Chen Y He Y Wang J Xing L., Automated classification of retinal optical coherence tomography volumes based on RepVGG: experimental validation for detection of dry age-related macular degeneration and diabetic macular edema. 2nd International Conference on Intelligent Computing and Human-Computer Interaction (ICHCI). IEEE, (2021), 299–2.

[27] Lin C Zheng Y Xiao X Lin J. CXR-RefineDet: single-shot refinement neural network for chest X-ray radiograph based on multiple lesions detection. J Healthcare Eng. (2022) 2022:1–11. doi: 10.1155/2022/4182191, PMID: 35035832 PMC8759881

[28] Trang K Nguyen AH TonThat L Vuong BQ. Improving RepVGG model with variational data imputation in COVID-19 classification. IAES Int J Artif Intell. (2022) 11:1278. doi: 10.11591/ijai.v11.i4.pp1278-1286

[29] Zhu K Wu J, Residual attention: a simple but effective method for multi-label recognition. Proceedings of the IEEE/CVF International Conference on Computer Vision (2021), Available at: https://openaccess.thecvf.com/content/ICCV2021/html/Zhu_Residual_Attention_A_Simple_but_Effective_Method_for_Multi-Label_Recognition_ICCV_2021_paper.html

[30] Soares E Angelov P Biaso S Froes MH Abe DK. SARS-CoV-2 CT-scan dataset: a large dataset of real patients CT scans for SARS-CoV-2 identification. MedRxiv (2020):2020–04. doi: 10.1101/2020.04.24.20078584,

[31] Zhao J Zhang Y He X Xie P, Covid-CT-dataset: a CT scan dataset about covid-19. preprint, arXiv:200313865.

